# Effect of Vitamin D Supplementation on Blood Pressure: A Systematic Review

**DOI:** 10.7759/cureus.81150

**Published:** 2025-03-25

**Authors:** Hannah John, Shrey Gondalia, Jitender Sharma, Gauri Shankar

**Affiliations:** 1 Medical Writing, Index Research International, Vadodara, IND; 2 Medicine, Jagruti Hospital, Jamnagar, IND; 3 Neurology, Base Hospital Delhi Cantt, New Delhi, IND

**Keywords:** blood pressure, cardiovascular health, hypertension, randomized controlled trials, systematic review, vitamin d

## Abstract

Hypertension is a major risk factor for cardiovascular disease and mortality. Emerging evidence suggests a potential role of vitamin D in blood pressure regulation through its influence on the renin-angiotensin-aldosterone system, endothelial function, and inflammation. However, findings from interventional studies remain inconsistent. This systematic review aims to evaluate the effect of vitamin D supplementation on systolic and diastolic blood pressure across various populations. A comprehensive literature search was conducted in PubMed, Cochrane Central, and ScienceDirect to identify randomized controlled trials and cohort studies investigating the impact of vitamin D on hypertension. Data extraction and risk of bias assessments were performed independently by two reviewers. The primary outcome was the change in systolic and diastolic blood pressure before and after vitamin D supplementation. In total, 12 studies met the inclusion criteria, with sample sizes ranging from 35 to 3,788 participants. The highest reduction in systolic blood pressure (-28.44 mmHg) and diastolic blood pressure (-7.38 mmHg) was observed in one study with 50,000 IU/week supplementation. Other studies reported reductions ranging from -0.5 to -4.5 mmHg for systolic blood pressure and -1 to -5 mmHg for diastolic blood pressure, with variability influenced by dosage and duration. Vitamin D supplementation demonstrates potential benefits in reducing blood pressure, particularly with higher doses over shorter durations. However, heterogeneity across studies warrants further research to establish optimal dosing strategies for hypertensive patients.

## Introduction and background

Hypertension, a leading global health concern, is a significant risk factor for cardiovascular disease, stroke, kidney failure, and premature mortality [1]. Despite advancements in antihypertensive therapies, the prevalence of hypertension continues to rise, necessitating alternative and adjunctive treatment strategies. In recent years, the role of vitamin D in blood pressure regulation has garnered increasing scientific interest due to its potential influence on vascular function, inflammation, and the renin-angiotensin-aldosterone system (RAAS) [2].

Vitamin D, a fat-soluble secosteroid, is primarily synthesized in the skin upon exposure to ultraviolet B radiation. It can also be obtained through dietary sources, including fortified foods and supplements [3]. The two major forms of vitamin D, cholecalciferol (vitamin D3) and ergocalciferol (vitamin D2), undergo hepatic conversion to 25-hydroxyvitamin D [25(OH)D], the main circulating form, followed by renal conversion to its biologically active form, 1,25-dihydroxyvitamin D [1,25(OH)2D]. Beyond its classical role in calcium and phosphate homeostasis, vitamin D has been implicated in a range of physiological processes, including immune modulation, endothelial function, and cardiovascular health [4].

Several epidemiological studies have suggested an inverse relationship between vitamin D levels and blood pressure, with vitamin D deficiency being associated with an increased risk of hypertension [5,6]. The proposed mechanisms underlying this association include vitamin D-mediated suppression of RAAS activity, enhanced endothelial function, reduced arterial stiffness, and anti-inflammatory effects [5]. Given the high prevalence of vitamin D deficiency worldwide, particularly in populations with limited sun exposure or darker skin pigmentation, its potential role in hypertension management has gained widespread attention [6].

Despite promising observational findings, interventional studies examining the effects of vitamin D supplementation on blood pressure have yielded inconsistent results. While some randomized controlled trials (RCTs) have reported significant reductions in systolic and diastolic blood pressure following vitamin D supplementation, others have found negligible or no effects [7]. These discrepancies may be attributed to variations in study design, baseline vitamin D status, dosage regimens, duration of intervention, and participant characteristics. Consequently, the need for a systematic review and meta-analysis to synthesize available evidence and provide a clearer understanding of the potential benefits of vitamin D in blood pressure regulation is paramount [8].

This systematic review aims to evaluate the impact of vitamin D supplementation on hypertension by analyzing data from RCTs and cohort studies conducted across diverse populations. By assessing changes in systolic and diastolic blood pressure pre- and post-intervention, this review seeks to elucidate the efficacy of vitamin D as a potential therapeutic strategy for hypertension management. Furthermore, the study explores possible sources of heterogeneity in findings and identifies key factors that may influence treatment outcomes.

Given the growing burden of hypertension and the potential role of vitamin D in cardiovascular health, elucidating the relationship between vitamin D supplementation and blood pressure regulation has significant clinical and public health implications. If proven effective, vitamin D supplementation could serve as an accessible, low-cost intervention to complement existing antihypertensive strategies. Therefore, this review provides a comprehensive analysis of current evidence, contributing to the ongoing discourse on the integration of nutritional interventions in hypertension management.

## Review

Methodology

Study Protocol

We adhered to the Preferred Reporting Items for Systematic Reviews and Meta-Analyses (PRISMA) guidelines for conducting and reporting this systematic review [9].

Data Sources and Searches

A comprehensive literature search was conducted in three major electronic databases, namely, PubMed, Cochrane Central, and ScienceDirect, to identify relevant studies assessing the effect of vitamin D on hypertension published after 2010. The search spanned from inception to the final date of retrieval. The search strategy incorporated Medical Subject Headings (MeSH) terms and keywords including “Vitamin D,” “hypertension,” “blood pressure,” and “clinical trial.” The records were restricted to human studies published in the English language.

Study Selection

Two independent reviewers screened the identified records by title and abstract to select eligible studies. Full-text articles were retrieved for studies that met the inclusion criteria. Studies were included if they met the following criteria: (1) RCTs, (2) investigated the association between vitamin D supplementation and blood pressure regulation, (3) included adult participants (≥18 years) from the general population without pre-existing secondary hypertension, and (4) reported blood pressure outcomes at baseline and post-intervention. Studies were excluded if they (1) assessed other vitamin D metabolites such as 1,25(OH)2D, (2) included pregnant women or specific disease populations, or (3) lacked sufficient data on blood pressure outcomes. Any discrepancies in the opinion were resolved through consensus discussion or consultation with a third reviewer.

Data Extraction

Data were extracted independently by two reviewers using a predefined data extraction template. The following details were collected: first author, publication year, study location, study design, sample size, intervention details (vitamin D dosage and duration), and blood pressure measurements before and after intervention. In cases where essential data were missing, corresponding authors were contacted to obtain additional information. If multiple follow-up points were available, only the longest follow-up duration was considered for analysis.

Risk of Bias Assessment

The methodological quality of included RCTs was assessed using the Cochrane Risk of Bias tool, which evaluates the following seven domains: random sequence generation, allocation concealment, blinding of participants and personnel, blinding of outcome assessment, incomplete outcome data, selective reporting, and other biases [10]. Studies were categorized as having a low, high, or unclear risk of bias based on these criteria. Two independent reviewers conducted the assessments, and disagreements were resolved by consensus.

Data Synthesis and Analysis

A qualitative synthesis of the included studies was performed to evaluate the impact of vitamin D supplementation on blood pressure regulation. The analysis focused on the direction and magnitude of changes in systolic and diastolic blood pressure, accounting for variations in study populations, intervention dosages, and treatment durations.

Results

**Figure 1:**
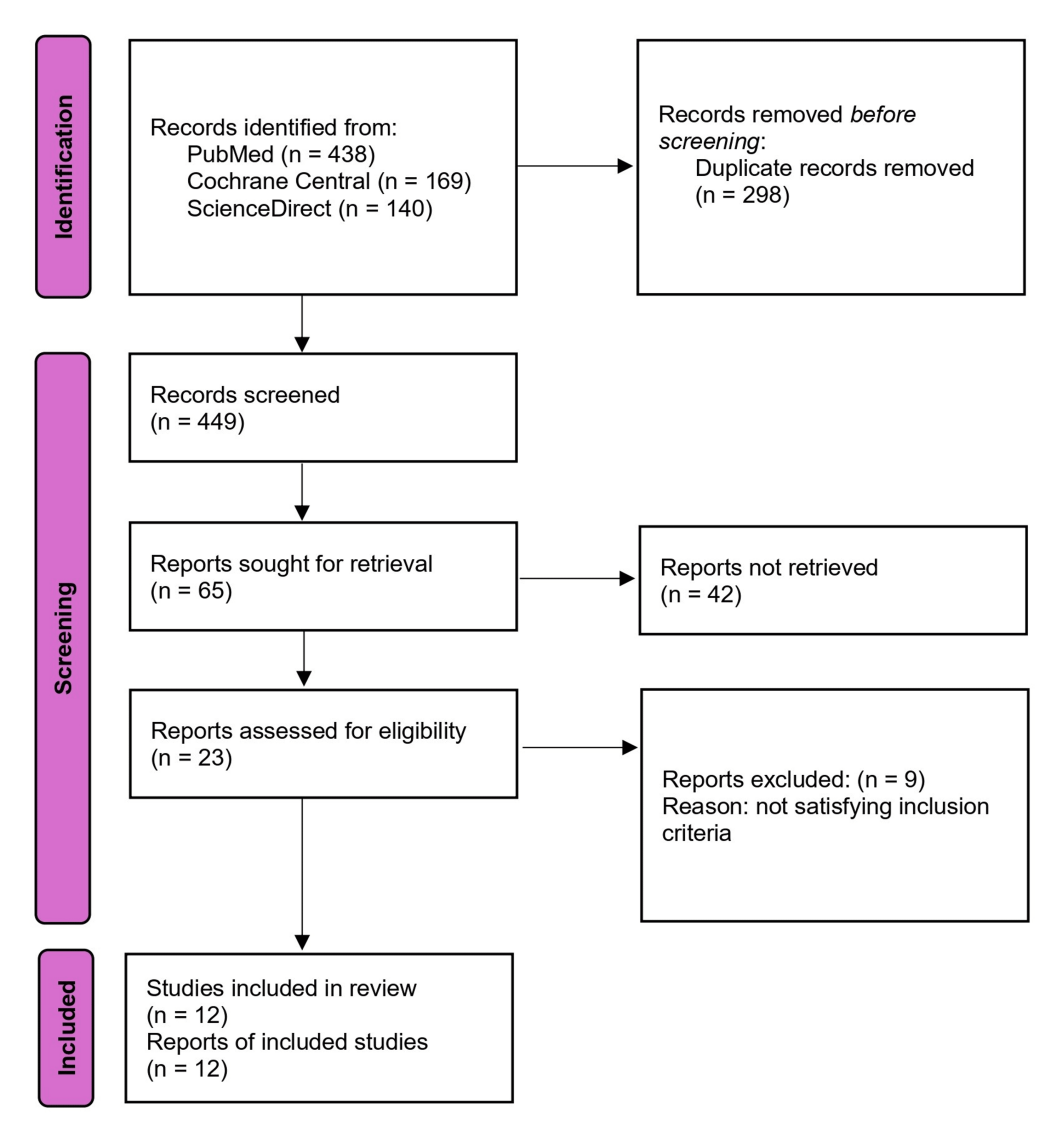
PRISMA flow diagram. PRISMA: Preferred Reporting Items for Systematic Reviews and Meta-Analysis

The initial search identified 747 studies from the databases. A total of 449 records were screened after the initial exclusion of the studies. Following an assessment of the titles and abstracts, 65 articles were selected for further consideration. Following that, 42 studies were eliminated based on the inclusion criteria. We screened 23 studies based on the inclusion and exclusion criteria. Finally, we selected 12 studies because of the unavailability of some data in the other studies. The process of selection of the studies is depicted in the PRISMA study selection diagram (Figure 1).

Study Characteristics

A total of 12 studies met the inclusion criteria, comprising RCTs conducted across various geographical regions, including the United States, Iran, Spain, Denmark, Australia, Austria, China, the United Kingdom, and Switzerland (Table 1). The sample sizes of these studies ranged from 35 to 3,788 participants, with varying intervention durations from 8 to 48 weeks. Vitamin D dosages also differed, spanning from 200 IU/day to 50,000 IU/week.

**Table 1:** Characteristics of the included studies. The values of systolic and diastolic blood pressure are written as mean ± SD or SE. SD: standard deviation; SE: standard error

Author	Country	Sample size	Vitamin D dosage and time	Before intervention		After intervention	
				Systolic blood pressure	Diastolic blood pressure	Systolic blood pressure	Diastolic blood pressure
Maki et al. (2011) [11]	USA	60	1,200 IU/day; 8 weeks	120.3 ± 1.9	74.1 ± 1.5	115.8 ± 1.8	71.9 ± 1.4
Salehpour et al. (2012) [12]	Iran	77	25 µg/day; 12 weeks	110.5 ± 17.5	67.9 ± 10.1	111 ± 11.3	70.2 ± 8.8
Toxqui et al. (2013) [13]	Spain	55	200 IU/day; 16 weeks	109.3 ± 10.4	69.2 ± 9.4	105.9 ± 9.1	66.6 ± 7.3
Wamberg et al. (2013) [14]	Denmark	52	7,000 IU/day; 26 weeks	135 ± 18	85 ± 10	129 ± 13	84 ± 11
Gagnon et al. (2014) [15]	Australia	35	2,000 IU/day; 24 weeks	120.8 ± 14	73.1 ± 10	118.4 ± 10.2	72.9 ± 8.2
Arora et al. (2015) [16]	USA	534	4,000 IU/day; 24 weeks	131 ± 10	82 ± 9	127 ± 10	77 ± 9
Pilz et al. (2015) [17]	Austria	200	2,800 IU/day; 8 weeks	131.4 ± 8.1	78.1 ± 7.5	130.3 ± 9.3	77.8 ± 8.2
Peng et al. (2016) [18]	China	3788	2,000 IU/day; 12 weeks	127.04 ± 14.86	81.95 ± 8.58	126.56 ± 14.4	81.75 ± 7.56
Tomson et al. (2017) [19]	UK	305	4,000 IU/day; 48 weeks	132.7 ± 21.1	78 ± 11.3	132.5 ± 1.43	77.2 ± 0.89
Bislev et al. (2018) [20]	Denmark	81	2,800 IU/day; 12 weeks	129	75 ± 9	128.7	75.9
Abderhalden et al. (2020) [21]	Switzerland	273	2,000 IU/day; 24 weeks	131.9 ± 12.4	79.1 ± 7.8	129.7 ± 12.1	77.2 ± 7.6
Sheik et al. 2020 [22]	Iran	208	50,000 IU/week; 8 weeks	157.29 ± 15.29	88.44 ± 9.10	128.85 ± 14.06	81.06 ± 4.38

Effects of Vitamin D Supplementation on Systolic Blood Pressure

Among the 12 included studies, 10 demonstrated a reduction in systolic blood pressure (SBP) following vitamin D supplementation. The reductions ranged from minimal changes (e.g., Pilz et al. 2015 [17], with a decrease of 1.1 mmHg) with a mean treatment effect (95% confidence interval (CI) of −0.4 (−2.8 to 1.9) mmHg (p = 0.712)) to more substantial reductions, such as in the study by Sheikh et al. 2020, where SBP decreased by 28.44 mmHg [22]. The greatest decrease was observed in studies where higher doses of vitamin D were administered over a shorter period, such as 50,000 IU/week for eight weeks in the study by Sheikh et al. 2020 [22].

The study by Maki et al. (2011) reported an SBP reduction of 4.5 mmHg (p = 0.09) after an eight-week intervention with 1,200 IU/day [11]. Toxqui et al. (2013) [13] reported a reduction of 3.4 mmHg in SBP after 16 weeks of intervention, while Wamberg et al. (2013) [14] found non-significant decrease of SBP (p = 0.11) in the vitamin D group from 135 ± 18 mmHg at baseline to 129 ± 13 mmHg after the intervention. Similarly, Arora et al. (2015) documented a decrease of 4 mmHg following a 24-week regimen of 4,000 IU/day [16]. A decrease was also observed in the study by Tomson et al. (2017), with a reduction of 0.2 mmHg after 48 weeks of 4,000 IU/day supplementation [19].

Effects of Vitamin D Supplementation on Diastolic Blood Pressure

Diastolic blood pressure (DBP) reductions were also noted across most studies, with the magnitude of change varying. The most significant decrease in DBP was observed in Sheikh et al. (2020), with a reduction of 7.38 mmHg [22]. In contrast, studies such as Salehpour et al. (2012) reported only a modest reduction of 2.3 mmHg [12]. Toxqui et al. (2013) [13] reported a significant reduction of 2.6 mmHg (p = 0.01) in DBP after 16 weeks of intervention. Several studies reported reductions in DBP of approximately 1-5 mmHg, including Maki et al. (2011) (-2.2 mmHg, p = 0.227) [11] and Wamberg et al. (2013) (-1 mmHg, p = 0.61) [14]. Abderhalden et al. (2020) showed 1.9 mmHg change in DBP post-intervention [21].

Overall, the findings suggest that vitamin D supplementation exerts a beneficial effect on blood pressure reduction, particularly in SBP. The extent of reduction appears to be influenced by the dosage, duration, and baseline blood pressure levels of the participants. Studies administering higher vitamin D doses over a shorter period generally observed a more significant impact. While the reductions in blood pressure were modest in some studies, the overall trend suggests a potential role for vitamin D in hypertension management. However, heterogeneity in study design and population characteristics warrants further investigation.

Critical Appraisal of Studies

We critically appraised our screened articles using the Cochrane Risk of Bias tool [10]. Table 2 presents a quality assessment of included studies, focusing on key indicators of study integrity.

**Table 2:** Quality assessment of the studies using the Cochrane Risk of Bias assessment tool.

Study	Random sequence generation	Allocation concealment	Blinding of participants and personnel	Blinding of outcomes assessment	Incomplete outcome data	Reporting bias	Other bias
Maki et al. (2011) [11]	Low	Low	Low	Low	Low	Low	Low
Salehpour et al. (2012) [12]	Low	Low	Low	Unclear	Low	Low	Low
Toxqui et al. (2013) [13]	Low	Low	Low	Low	High	Low	Low
Wamberg et al. (2013) [14]	Low	Low	Low	Low	Low	Low	Low
Gagnon et al. (2014) [15]	Low	Low	Low	Unclear	Low	Low	Low
Arora et al. (2015) [16]	Low	Low	Low	Low	Low	Low	Low
Pilz et al. (2015) [17]	Low	Low	Low	Low	Low	Low	Low
Peng et al. (2016) [18]	Low	High	High	High	High	Low	Low
Tomson et al. (2017) [19]	Low	Low	Low	Low	Low	Low	Low
Bislev et al. (2018) [20]	Low	Low	Low	Unclear	High	Low	Low
Abderhalden et al. (2020) [21]	Low	Low	Low	Unclear	Low	Low	Low
Sheik et al. (2020) [22]	Low	Low	Low	Low	Low	Low	Low

Discussion

This systematic review evaluates the impact of vitamin D supplementation on blood pressure regulation, with a particular focus on its effects on systolic and diastolic blood pressure across different populations and intervention strategies. The findings suggest that vitamin D supplementation has the potential to lower blood pressure, although the magnitude of reduction varies depending on dosage, duration, baseline characteristics, and study design.

The overall trend observed in this review indicates that vitamin D supplementation contributes to a reduction in both systolic and diastolic blood pressure. Among the 12 studies included, the majority reported reductions in SBP, with decreases ranging from 1.1 mmHg (Pilz et al., 2015) [17] to 28.44 mmHg (Sheikh et al., 2020) [22]. Similarly, DBP reductions were also reported, 2.3 mmHg by Salehpour et al. (2012) [12]. Notably, the greatest reductions in blood pressure were observed in studies with higher vitamin D dosages administered over shorter periods, suggesting that both dose and intervention duration play a crucial role in determining efficacy [22]. Significant reduction (p = 0.017) in mean SBP from 108.6 ± 8.2 mmHg at eight weeks to 105.9 ± 9.1 mmHg after 16 weeks was reported by Toxqui et al. (2013) [13] in the D-fortified group. The same authors also found a significant reduction (p = 0.01) in mean DBP from 69.2 ± 9.4 mmHg of baseline value to 66.6 ± 7.3 mmHg after 16 weeks in the D-fortified group. Contrary to the findings of Toxqui et al. (2013) [13], Wamberg et al. (2013) [14] reported non-significant decrease of SBP (p = 0.11) in the vitamin D group from 135 ± 18 mmHg at baseline to 129 ± 13 mmHg after the intervention and also non-significant reduction in DBP (p = 0.61) in the vitamin D group from 85 ± 10 mmHg at baseline to 84 ± 11 mmHg after the intervention. Similarly, a non-significant effect of vitamin D supplementation on 24-hour SBP with a mean treatment effect (95% CI of −0.4 (−2.8 to 1.9) mm Hg (p = 0.712) was reported by Pilz et al. (2015) [17] between the vitamin D and placebo groups.

The study by Sheikh et al. (2020), which administered 50,000 IU/week for eight weeks, demonstrated the most significant SBP reduction of 28.44 mmHg and a DBP reduction of 7.38 mmHg [22]. In the first and second months following the intervention, the effect of vitamin D supplementation on SBP was statistically significant (p = 0.004 and p = 0.024, respectively). In the first month following the intervention, vitamin D supplementation had a statistically significant effect on DBP (p = 0.046), but not in the second month (p = 0.885). This finding suggests that high-dose supplementation over a short duration may yield greater blood pressure reductions, potentially due to the rapid correction of vitamin D deficiency. Other studies that used moderate doses over longer durations, such as Arora et al. (2015) with 4,000 IU/day for 24 weeks, also showed clinically relevant reductions in SBP (4 mmHg) [16]. These results align with previous research suggesting that vitamin D exerts antihypertensive effects, though the degree of response may depend on baseline vitamin D status and underlying metabolic factors [23].

The biological mechanisms underlying the relationship between vitamin D and blood pressure regulation are multifaceted. Vitamin D is believed to influence blood pressure through several pathways, including its effects on RAAS, endothelial function, inflammation, and calcium homeostasis [2]. One of the most widely studied mechanisms is the ability of vitamin D to modulate RAAS. Vitamin D has been shown to inhibit renin expression, leading to a downregulation of angiotensin II production and a subsequent reduction in vasoconstriction and sodium retention. This mechanism could explain why vitamin D supplementation leads to reductions in blood pressure, particularly in individuals with vitamin D deficiency, where RAAS activity may be upregulated [24].

Vitamin D is known to enhance endothelial function by increasing nitric oxide production and reducing oxidative stress. Improved endothelial function leads to better vasodilation and arterial compliance, which could contribute to the observed reductions in blood pressure [25]. Chronic low-grade inflammation is a known contributor to hypertension. Vitamin D has demonstrated anti-inflammatory properties by reducing pro-inflammatory cytokines such as interleukin-6 and tumor necrosis factor-alpha. The anti-inflammatory effects of vitamin D may be particularly relevant in populations with metabolic syndrome or obesity, where inflammation contributes to increased blood pressure [26]. The study by Arora et al. (2015), which showed a 4 mmHg reduction in SBP, supports this hypothesis, as its population included individuals with prehypertension or hypertension [16].

While the majority of studies reported blood pressure reductions, some demonstrated only modest effects. For example, Salehpour et al. (2012) observed a minimal change in SBP (−0.5 mmHg) and a 2.3 mmHg decrease in DBP after 12 weeks of supplementation with 25 μg/day [12]. Non-significant change in mean SBP (p = 0.3) and DBP (p = 0.207) was observed between the vitamin D group and the placebo group after 12 weeks of intervention [12]. Similarly, Pilz et al. (2015) reported a reduction of only 1.1 mmHg in SBP [17]. Individuals with vitamin D deficiency are more likely to experience greater reductions in blood pressure following supplementation compared to those with sufficient baseline levels. Genetic polymorphisms in vitamin D receptor genes may influence individual responses to supplementation. Moreover, variations in baseline blood pressure and hypertension prevalence across different ethnic populations could contribute to differing results [27]. Shorter intervention periods showed more changes in SBP and DBP compared to longer duration interventions, as noticed by Tomson et al. (2017) [19]. Variability in measurement techniques, participant characteristics, and adherence to supplementation regimens could influence the observed outcomes.

The findings of this systematic review have important implications for the clinical management of hypertension and public health strategies aimed at cardiovascular risk reduction. Given the generally favorable safety profile of vitamin D supplementation, it could serve as an adjunctive therapy for individuals at risk of hypertension, particularly those with low baseline vitamin D levels. However, the optimal dosing regimen remains to be determined, as higher doses appear to be more effective in reducing blood pressure but may raise concerns about potential adverse effects with prolonged use.

From a public health perspective, addressing vitamin D deficiency through dietary supplementation, fortified foods, or lifestyle modifications such as increased sun exposure could be a cost-effective strategy to reduce the hypertension burden globally. This is particularly relevant in regions with high prevalence of vitamin D deficiency, where supplementation programs may yield substantial cardiovascular benefits [28].

Despite the promising findings, this review has a few limitations. Heterogeneity in study designs, participant characteristics, and intervention protocols limits direct comparability between studies. While most studies reported reductions in blood pressure, the clinical significance of these reductions varies, and longer-term outcomes remain unclear. Potential confounding factors such as dietary habits, physical activity, and concurrent medication use were not uniformly accounted for in all studies.

## Conclusions

This study highlights the potential role of vitamin D supplementation in reducing blood pressure, particularly SBP, in hypertensive and prehypertensive individuals. While significant heterogeneity exists across studies, the overall trend suggests a beneficial effect, with higher doses and shorter intervention periods demonstrating the greatest reductions. Given the global burden of hypertension and the widespread prevalence of vitamin D deficiency, further well-designed clinical trials are needed to establish definitive guidelines for vitamin D supplementation in hypertension management.
